# A Novel Serum-Free Triculture Model of Glioblastoma, Astrocytes, and Macrophages

**DOI:** 10.3390/ijms26199335

**Published:** 2025-09-24

**Authors:** Hasan Alrefai, Lauren C. Nassour-Caswell, Manoj Kumar, Benjamin Lin, Taylor L. Schanel, Nicholas J. Eustace, Jianqing Zhang, Christian T. Stackhouse, Nayonika Mukherjee, Patricia H. Hicks, Joshua C. Anderson, Christopher Ryan Miller, Christopher D. Willey

**Affiliations:** 1 Department of Radiation Oncology, Heersink School of Medicine, The University of Alabama at Birmingham, Birmingham, AL 35233, USA; ha0011@uab.edu (H.A.); nassour@uab.edu (L.C.N.-C.); manokuma4575@uabmc.edu (M.K.); tschanel@uab.edu (T.L.S.); jianqing@uab.edu (J.Z.); hicksp@uab.edu (P.H.H.); janders7@uab.edu (J.C.A.); 2 Division of Neuropathology, Department of Pathology, Heersink School of Medicine, The University of Alabama at Birmingham, Birmingham, AL 35233, USAryanmiller@uabmc.edu (C.R.M.); 3 Department of Radiation Oncology, City of Hope National Medical Center, Duarte, CA 91010, USA; neustace@coh.org; 4 Department of Pediatrics, Division of Hematology & Oncology, Duke University Medical Center, Durham, NC 27708, USA; ctstackh@gmail.com; 5 Division of Pharmacology and Toxicology, Department of Pathology, Heersink School of Medicine, The University of Alabama at Birmingham, Birmingham, AL 35233, USA; nmukherj@uab.edu

**Keywords:** glioblastoma, astrocytes, tumor-associated macrophages, triculture model system, patient-derived xenograft

## Abstract

Glioblastoma (GBM) is the most common and deadly primary brain tumor in adults. While in vitro patient-derived xenografts (PDX) lines are useful for studying GBM, they often exclude astrocytes and macrophages, which contribute significantly to tumor growth, invasion, and chemoradioresistance. Integrating these cells into tumor models is difficult due to their need for serum, which triggers GBM-PDX lines to lose their stem-like properties. The aim of this study was to develop a serum-free triculture model of GBM-PDX lines, normal human astrocytes (NHAs), and macrophages. Serum-free media alternatives were formulated for NHAs and identified for THP-1 macrophages, then combined with GBM PDX media to establish “PSX,” an experimental maintenance media. Cells were transitioned to serum-free media alternatives and functionally assessed through several parameters unique to each cell type. In addition to assessing GBM “stemness,” a custom 350-gene NanoString chip was used to assess differential gene expression in monocultured PDX cells versus PDX cells exposed to NHAs and macrophages. PSX maintained canonical function in astrocytes and macrophages while preserving the stem-like properties of GBM-PDX cells. Tri-culturing all three cells increased the expression of stemness-associated transcription factors and increased the expression of genes related to stemness and hypoxia in GBM cells. GBM PDX cells exposed to NHAs and macrophages in direct triculture exhibit increases in markers of stemness and hypoxia. These findings suggest that the serum-free triculture model presented herein may better recapitulate the tumoral heterogeneity of GBM in vitro, providing a novel model to utilize in current research.

## 1. Introduction

Glioblastoma (GBM) is a uniformly lethal primary brain tumor that is resistant to traditional therapies. Despite maximally safe surgical resection, radiation therapy, and temozolomide, the tumor invariably recurs, resulting in a dismal median survival of 15–18 months [1]. GBM is highly heterogeneous, hosting several different cell types within and surrounding the tumor [2]. It is believed tumor recurrence is due to a subset of highly resistant GBM cells localized to the perivascular niche called brain tumor-initiating cells (BTICs). BTICs also recruit astrocytes and peripheral monocytes through a variety of signaling mechanisms. BTICs promote differentiation and polarization of these cells into tumor-supportive cells that remodel the tumor microenvironment (TME) to facilitate tumor growth, invasion, vascularization, chemoradioresistance, and immunosuppression [3,4,5]. To develop a better preclinical model, one needs to more accurately replicate the complex interactions between the major cell types comprising the GBM TME.

Astrocytes account for up to half of the total cells in the mammalian brain and are one of the dominant cell populations in the TME [6,7]. Normally, these glial cells function to maintain the blood–brain barrier (BBB), support neural cell development, and distribute energetic substrates, such as glutamate, throughout the brain [8,9]. Astrocytes can become activated to one of many states in response to a variety of insults, such as infection, stroke, and trauma [7]. Activated astrocytes function to repair the normal brain. However, their frequent participation in normal brain processes makes them a vulnerable target for recruitment to the TME, especially by neoplastic BTICs. BTICs are known to recruit and modify astrocytes towards a tumor-supportive, reactive phenotype [7,10,11,12,13]. These cells are referred to as tumor-associated astrocytes (TAAs) and are known to support GBM growth and progression by promoting the formation of vast networks of different cell types [7,13]. Modeling astrocytes without serum not only benefits co-culture studies but also represents the canonical astrocyte function and morphology seen in vivo [14,15]. Importantly, astrocytes are typically “resting” unless faced with a biological insult [14,15]. Though astrocyte “reactivity” can be considered an umbrella term, it is known that fetal bovine serum (FBS) naturally induces a more reactive phenotype regardless of the physiological scenario, changing the morphology and ability to assess the transition from resting to reactive [11,12,14]. A serum-free culture environment would permit for better assessment of astrocytic influence, especially in the context of serum-sensitive tumors such as glioma.

BTICs also function to recruit and differentiate peripheral monocytes into tumor-supportive macrophages by a variety of signaling mechanisms. These macrophages are collectively known as tumor-associated macrophages (TAMs) and significantly influence GBM biology. TAMs are the most abundant non-cancerous cell type in the TME, accounting for up to 40% of tumor mass and over 70% of the immune cells [16]. The redundancy of immunosuppressive ligands expressed by TAMs contributes to the widespread failure of anti-PD-1 and anti-PD-L1 therapy in GBM in clinical trials. As such, there is growing interest in therapeutically targeting TAMs for reprogramming. While there has been drastic improvement in the modeling of gliomas, there is a dearth of high-fidelity TAM models. Traditionally, macrophage polarization states have been classified as resting (M0), pro-inflammatory (M1), or anti-inflammatory (M2a-d). Classically, M1 macrophages have increased expression of CD80 and HLA-DR, and produce pro-inflammatory cytokines such as TNFα and IL-1ß [17]. M2 macrophages have increased expression of CD163 and CD206 (MRC1), and produce anti-inflammatory, ECM remodeling, and pro-angiogenic cytokines [17,18]. Interleukins such as IL-4, IL-10, and IL-13 are frequently used to polarize macrophages towards an M2-like state to mimic TAMs in vitro [19]. Unfortunately, this classic method of macrophage polarization is not representative of TAM function in vivo. Additionally, several single-cell studies have revealed several subpopulations of TAMs which are highly heterogeneous in their function and cell-surface marker expression, further confirming that the M1 vs. M2 dichotomy is not suitable for characterizing TAMs [16,20,21,22]. Together, these studies highlight the necessity of developing higher-fidelity model systems to accurately model TAM function in vitro.

Before the emergence of advanced patient-derived models of GBM, immortalized GBM cell lines, such as U87MG and U251MG, were derived from GBM patients in the 1960s and have been maintained in serum ever since [23,24]. Immortalized GBM cell lines are homogenous in nature and are therefore advantageous for molecular mechanistic studies; however, these lines fail to recapitulate the tumoral heterogeneity of human GBM and often fail to be reliable therapeutic response models. In place of immortalized and murine glioma cell lines, PDX BTICs have gained increased popularity over the years based on their stem-like properties and more representative genetic and phenotypic profiles [24]. PDX cell lines can grow as neurospheres and are maintained by serially passaging cells in mice subcutaneously or orthotopically, the latter being the most recapitulative of the TME [24]. Despite this being an improved GBM model, the necessary use of immunocompromised mice to passage these cells compromises the ability to study adaptive immune interactions. Furthermore, PDX cells are typically grown in monoculture. As previously mentioned, GBM comprises a plethora of cancerous and non-cancerous cell types that contribute to tumor chemo- and radio-resistance.

The overall complexity of TME heterogeneity highlights the importance of developing a physiologically relevant GBM model capable of high-throughput assessment. Unfortunately, most GBM models lack normal human astrocytes (NHAs) and macrophages due to challenges in maintaining three distinct cell types with varying cell culture requirements. Tang et al. developed a three-dimensional tetra-culture model of GBM that incorporated GBM PDX cells, NHAs, IL4/13-polarized THP-1 macrophages, and neural progenitor cells (NPCs) [25]. While their method presents a novel and interesting approach to modeling GBM, the incorporation of serum to support the growth of all four cell types convolutes the ability to substitute stem-like cells into the model. Though this study was an instrumental stride in the field of modeling, we sought to formulate a serum-free version of maintenance media capable of supporting all three cell types simultaneously while maintaining GBM PDX stemness and promoting growth. In this study, we discuss serum-free media formulations that support the long-term growth of NHAs and THP-1 monocytes while preserving canonical function.

## 2. Results

### 2.1. Methodology for Culturing PDX BTICs, NHAs, and THP-1 Monocytes in a Serum-Free Maintenance Media

**Figure 1:**
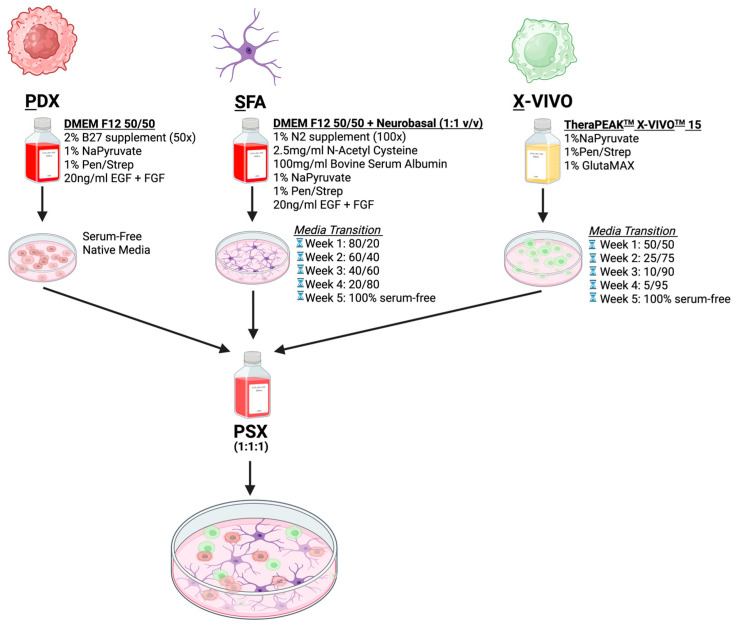
Triculture modeling methodology. Schematic demonstrating formulation of PDX, SFA, and X-VIVO media and serum-free media transition schedules. Created in BioRender. Nassour, L. (2025) https://BioRender.com/i1g4x8b (accessed on 14 September 2025).

One of the largest obstacles in developing a serum-free triculture model is identifying serum-free alternatives for non-cancerous cells that are traditionally cultured in serum-containing media. To mitigate this, we formulated a media alternative for NHAs, termed serum-free astrocyte (SFA) media, and identified a commercially available (X-VIVO) serum-free alternative for THP-1 monocytes. **P**DX media was combined with **S**FA and **X**-VIVO media at equal ratios to establish PSX, a serum-free triculture media (Figure 1). PDX cells, NHAs, and THP-1 cells were transitioned over several weeks into their serum-free alternatives, respectively (Figure 1). Cells were grown in the experimental media conditions for at least 1 week prior to assessing various validation metrics.

### 2.2. SFA and PSX Serum-Free Media Alternatives Support Canonical Function in NHAs

**Figure 2:**
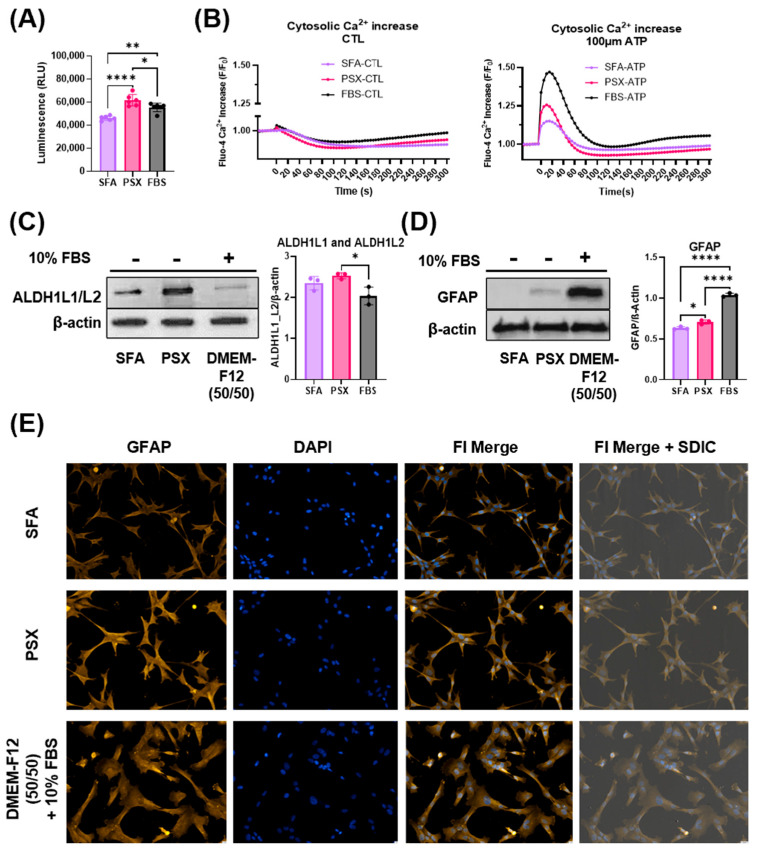
Serum-free astrocyte media alternatives, SFA and PSX media, promote canonical astrocyte morphology and function (*n* = 6). (**A**) Cell viability effects of different media on NHAs grown for seven days. (**B**) Cytosolic Ca^2+^ levels of NHAs at baseline (CTL) compared to ATP stimulation (*n* = 5). (**C**) Levels of astrocytic markers, ALDH1L1/L2, when grown in respective media for 7 days. (**D**) Levels of reactive astrocyte marker, GFAP, when grown in different media (*n* = 3). (**E**) Representative images of astrocytic morphology when grown in respective media. All experiments were performed in at least three technical replicates and repeated independently three times. (* *p* < 0.05, ** *p* < 0.01, **** *p* < 0.0001).

We assessed the growth of NHAs after 7 days in native DMEM:F12 50/50 supplemented with 10% FBS, SFA, and PSX media. NHAs grown in SFA had slower growth compared to native 10% FBS media (*p* = 0.0019) (Figure 2A). Interestingly, there was a slight increase in growth of NHAs in PSX relative to native FBS-containing media and SFA (*p* = 0.0304 and *p* < 0.0001, respectively) (Figure 2A). Astrocytes regularly participate in calcium signaling; therefore, we investigated this phenomenon after astrocytes were grown in the experimental media. Upon comparing SFA, PSX, and 10% FBS control groups 5 s after administering phosphate-buffered saline (PBS), we observed a moderate but significant increase in the 10% FBS group compared to the serum-free alternative media but no difference between SFA and PSX (5 s: SFA vs. 10% FBS: *p* = 0.0250; PSX vs. 10% FBS: *p* = 0.0022; SFA vs. PSX: *p* = 0.9909) (Figure 2B). We observed this same trend upon addition of 100 µM ATP stimulus, in which 10% FBS-containing media was significantly increased compared to SFA and PSX. We observed the peak Ca^2+^ increase following 100µM ATP between 15 and 30 s, with no difference between SFA and PSX but a significant difference between 10% FBS and SFA and PSX (15 s: SFA vs. 10% FBS: *p* = 0.0159; PSX vs. 10% FBS: *p* = 0.0350; SFA vs. PSX: *p* = 0.4765) (Figure 2B). Aldehyde dehydrogenase 1 family member L1 and L2 (ALDH1L1/L2) are common combination astrocyte-specific markers, therefore we chose to quantify these markers when astrocytes were grown in different media conditions. Western blot analysis revealed that ALDH1L1/L2 levels were upregulated in astrocytes grown in PSX relative to FBS (*p* = 0.0228); however, there was no significant difference in ALDH1L1/L2 levels between astrocytes grown in PSX vs. SFA (*p* = 0.4104) or SFA vs. FBS (*p* = 0.1230) (Figure 2C). Glial fibrillary acidic protein (GFAP) is upregulated in astrocytes when they adopt a reactive state. We took advantage of this property to measure the levels of this protein in astrocytes grown in DMEM-F12 supplemented with 10% FBS, SFA, or PSX. Western blot analysis revealed NHAs exposed to 10% FBS possessed significantly higher levels of GFAP relative to both SFA and PSX (vs SFA: *p* < 0.0001; vs. PSX: *p* < 0.0001) (Figure 2D). Additionally, astrocytes grown in PSX had slightly increased GFAP relative to those grown in SFA (*p* = 0.0140) (Figure 2D). Representative immunofluorescence staining revealed SFA- and PSX-exposed NHAs retain the star-like morphology unique to astrocytes with increased branching processes (white arrows), while NHAs exposed to 10% FBS adopt a less-defined shape with thick membranous connections (white arrows) (Figure 2E).

### 2.3. X-VIVO Media Supports the Serum-Free Growth of THP-1 Monocytes While Preserving Function

**Figure 3:**
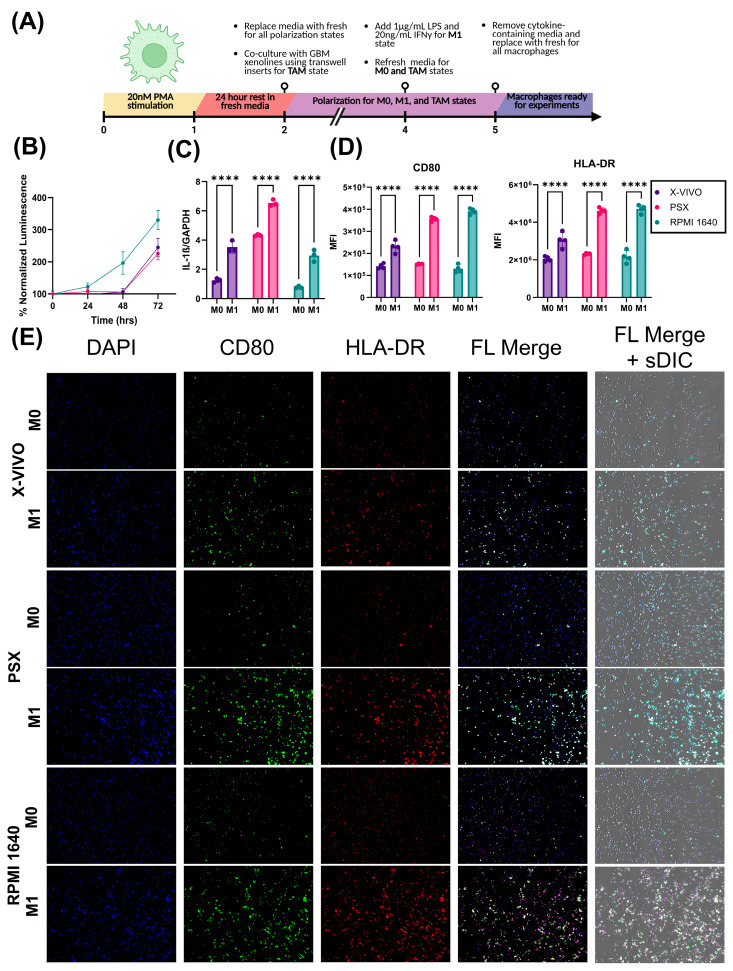
Serum-free media alternatives for macrophages, X-VIVO and PSX, support canonical macrophage function. (**A**) Polarization schematic for differentiating THP-1 monocytes into macrophages and polarizing them towards an M0, M1, or TAM state. Created in BioRender. Alrefai, H. (2025) https://BioRender.com/n4nyuee (accessed on 14 September 2025). (**B**) Percent normalized luminescence values of THP-1 monocytes when grown in native serum-containing RPMI1640, serum-free X-VIVO, or experimental maintenance PSX media over 72 h (*n* = 8). (**C**) Normalized expression of IL-1B assessed through dPCR (*n* = 3). (**D**) Quantification of MFI of CD80 and HLA-DR when THP-1 cells were grown in respective media (*n* = 4). (**E**) Representative images of THP-1 monocytes in M0 or M1 phase probed for CD80 and HLA-DR. All experiments were performed in at least three technical replicates and repeated independently twice (**** *p* < 0.0001).

Next, we assessed the growth dynamics of THP-1 monocytes when grown in native RPMI1640 supplemented with 10% FBS, X-VIVO, and PSX media. After 72 h of growth in each medium, cell growth was assessed using the luminescent cell viability assay CellTiter-Glo. There was no difference between X-VIVO and PSX media (*p* = 0.0878) (Figure 3B). Contrastingly, THP-1s grown in RPMI 1640 supplemented with 10% FBS demonstrated a significant increase in growth compared to both serum-free alternatives, X-VIVO and PSX (RPMI vs. X-VIVO: *p* < 0.0001; RPMI vs. SFA, *p* < 0.0001) (Figure 3B). We next sought to determine whether serum-free THP-1-derived macrophages would behave as expected in response to cytokine stimulation. THP-1 monocytes were differentiated into macrophage-like cells and polarized towards an M1-like state by stimulation with LPS and IFNγ (Figure 3A). THP-1 macrophages grown in serum-free and serum-containing conditions both demonstrated significant increases in IL-1β expression relative to their M0 controls (X-VIVO: *p* < 0.0001, PSX: *p* < 0.0001; RPMI: *p* < 0.0001) (Figure 3C). Additionally, we observed an increase in the mean fluorescent intensity of the classical M1-associated markers CD80 (X-VIVO: *p* < 0.0001, PSX: *p* < 0.0001; RPMI: *p* < 0.0001) and HLA-DR (X-VIVO: *p* < 0.0001, PSX: *p* < 0.0001; RPMI: *p* < 0.0001) in both serum-containing and serum-free conditions relative to their M0 controls (Figure 3D,E).

### 2.4. PSX Media Supports Serum-Free Growth and Maintains the Stemness Properties of JX14P

**Figure 4:**
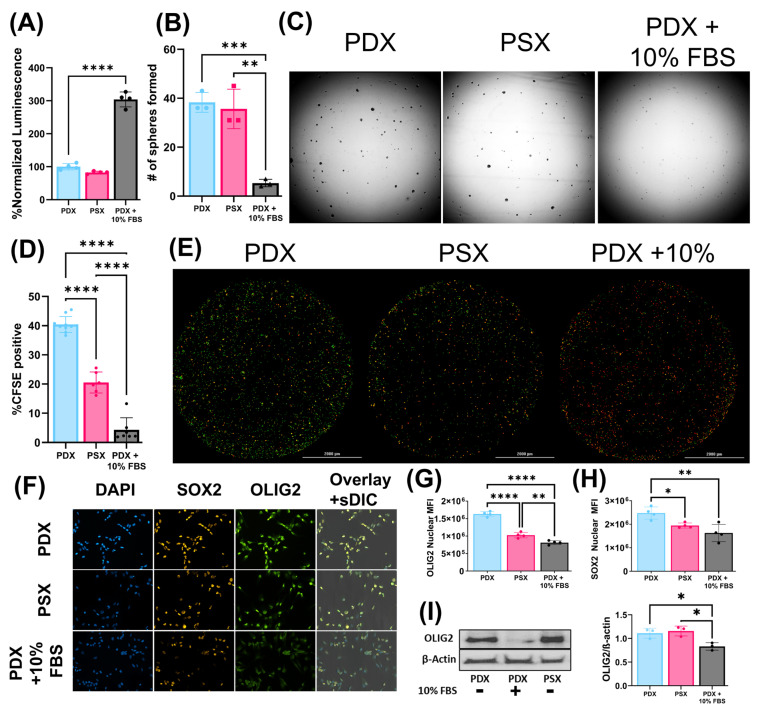

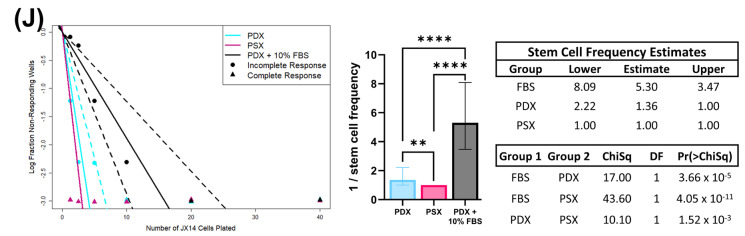
PSX media supports the serum-free growth and maintains the stemness properties of JX14. (**A**) Relative viability of JX14 when grown in native PDX media, PSX, or PDX media supplemented with 10% FBS (*n* = 4). (**B**) Quantification of the number of spheres formed in different media conditions when cells were grown in respective media for 7 days and then replated in standard PDX media for assessment of sphere-formation capacity (*n* = 3). (**C**) Representative images of spheres formed. (**D**) Percentage of cells that retained CFSE following growth in respective media (*n* = 6–10). (**E**) Representative images of NLS-mCherry-labeled cells (red) that retained the CFSE dye (green) (**F**) Representative images of JX14 probed with stemness markers SOX2 and OLIG2. (**G**) Quantification of OLIG2 nuclear MFI in respective media (*n* = 4). (**H**) Quantification of SOX2 nuclear MFI in respective media (*n* = 4). (**I**) Representative Western blot of OLIG2 protein concentrations with quantification in JX14 after growth in respective media for 7 days (*n* = 3). (**J**) Extreme limiting dilution assay (ELDA) performed on JX14 when grown in respective media (*n* = 10). Dashed lines represent 95% confidence intervals. All experiments were performed in at least three technical replicates and repeated independently three times (* *p* < 0.05, ** *p* < 0.01, *** *p* < 0.001, **** *p* < 0.0001).

After establishing that serum-free media (SFA and X-VIVO) and their combinations (PSX) could be used to grow non-tumor cells, we sought to investigate the effect PSX media had on the GBM PDX line JX14P. JX14P was grown in either PDX media, PDX media supplemented with 10% FBS, or PSX media. JX14P was first grown for seven days in the following media: native PDX, PDX + 10% FBS, and PSX. JX14P grown in PDX + 10% FBS exhibited the highest growth after one week (10% FBS vs. PDX: *p* < 0.0001; 10% FBS vs. PSX: *p* < 0.0001) while there was no significant difference found between the serum-free native PDX and PSX media (*p* = 0.2481) (Figure 4A).

Next, we sought to characterize the effects of PSX on the stem-like properties of JX14P as monocultures. We assessed the sphere-forming capabilities of JX14P after being grown in the respective media for one week prior. Spheres were selected and counted based on the size criteria of 40–150 µm. Similarly to the growth assay, there was no significant difference in sphere-forming capacity of JX14P grown in native PDX media compared to PSX (*p* > 0.9999) (Figure 4B,C). However, JX14P grown in 10% FBS had significantly diminished sphere-forming capacity compared to both serum-free media conditions (10% FBS vs. PDX: *p* = 0.0604; 10% FBS vs. PSX: *p* = 0.0641) (Figure 4B,C). Building upon this, we evaluated rates of cell division using carboxyfluorescein succinimidyl ester (CFSE) dye. Interestingly, cells grown in PSX media had slightly decreased rates of CFSE retention relative to those grown in PDX media; however, both media had significantly higher percentages of CFSE retention relative to those grown in 10% FBS (Figure 4D,E). We sought to further characterize the stem-like properties of JX14P monocultures by quantifying expression of certain stemness-associated transcription factors, such as oligodendrocyte transcription factor 2 (OLIG2) and sex determining region y-box 2 (SOX2) transcription factor staining. The nuclear median fluorescent intensity (MFI) of OLIG2 was significantly higher in native PDX and PSX relative to cells grown in 10% FBS (10% FBS vs. PDX: *p* <0.0001; 10% FBS vs. PSX: *p* = 0.0052) (Figure 4F,G). Interestingly, there was a mild decrease in OLIG2 expression in the PSX group relative to the PDX group (*p* < 0.0001). Similar trends were observed using a matched radiation-therapy selected GBM PDX line, JX14P-RT (Figure S1A,B). The nuclear MFI of SOX2 was significantly higher in PDX than other groups. There was no significant difference in the expression of SOX2 between PSX and FBS-containing groups (Figure 4F,H). Interestingly, there was no significant difference between PSX and any other group while using the PDX line JX14P-RT; however, cells grown in PDX media still had significantly higher SOX2 nuclear MFI than those grown in media supplemented with FBS (Figure S1A,C). These findings were further validated by Western blot analysis, which found no difference in native PDX media compared to PSX (*p* = 0.8355) (Figure 4I). Furthermore, 10% FBS conditions demonstrated significantly lower OLIG2 levels compared to both serum-free alternatives (10% FBS vs. PDX: *p* = 0.0277; 10% FBS vs. PSX: *p* = 0.0144) (Figure 4I). These results were consistent when performed with JX14P-RT (Figure S1D) and additional PDX lines (Figure S2). Finally, we performed the gold-standard, in vitro assay assessing stemness capabilities, the extreme limiting dilution assay (ELDA). JX14P cells grown in native PDX media were estimated to require 1.36 cells to form a colony. Comparably, those grown in PSX were estimated to require 1.00 cell to form a colony. When grown in 10% FBS-supplemented media, 5.30 cells were estimated to be necessary to form a colony (Figure 4J). Although the stem-cell frequency differed slightly, JX14P-RT exhibited a similar trend where PDX and PSX groups had similar levels of stem-cell frequency and those grown in FBS had significantly lower stem-cell frequencies (Figure S1E). Collectively, these results suggest that PSX media is sufficient to support the serum-free growth of NHAs, macrophages, and GBM PDX lines while preserving NHA and macrophage function as well as GBM PDX BTIC properties.

### 2.5. PDX Cells Grown in Triculture Demonstrate Increases in Stemness-Associated Markers

**Figure 5:**
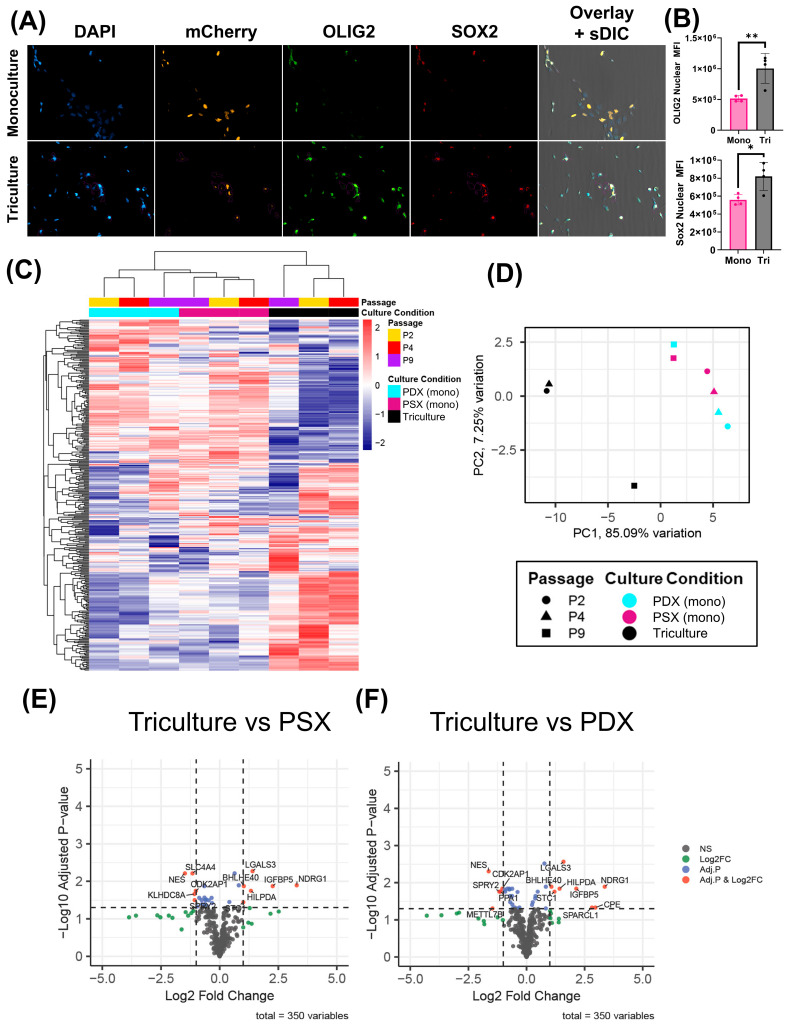
JX14 cells exhibit increased stemness markers when grown in triculture. (**A**) Representative images of NLS-mCherry JX14 cells when stained with OLIG2 and SOX2 (20×/0.5 NA). (**B**) Quantification of OLIG2 and SOX2 nuclear MFI for NLS-mCherry grown in direct triculture compared to monoculture (*n* = 4). (**C**) Heatmap of all 350 genes present on the Custom NanoString Chip for JX14 grown in PDX (monoculture), PSX (monoculture), and PSX (triculture). (**D**) Principal component analysis of JX14 grown in PDX (monoculture), PSX (monoculture), and PSX (triculture) *(n* = 3). (**E**) Volcano plot visualization of differentially expressed genes between JX14 grown in triculture vs. those grown in PSX media. (**F**) Volcano plot visualization of differentially expressed genes between JX14 grown in triculture vs. those grown in PDX media (* *p* < 0.05, ** *p* < 0.01).

We next sought to combine the cell types into a single model. THP-1-derived TAMs were co-cultured alongside NHAs and an NLS-mCherry-labeled JX14P. We then performed an OLIG2 and SOX2 staining experiment. Cells were gated based on NLS-mCherry positivity. Interestingly the nuclear MFI of both OLIG2 and SOX2 were significantly increased in triculture conditions relative to monoculture conditions (SOX2: *p* = 0.0271; OLIG2: *p* = 0.0201) (Figure 5A,B). To elucidate any variation in gene expression between the different culture conditions, we utilized a custom 350-gene NanoString chip to investigate JX14P grown as a monoculture in PDX media and PSX media, as well as in triculture indirectly exposed to astrocytes and macrophages in PSX media. Initial data analysis revealed strong sample variations correlated to culture passage number. To account for variations due to cell culture passage effects, we performed batch correction and included passage as a covariate in our design formula to account for any differences in gene expression due to prolonged in vitro culture. Unsupervised hierarchal clustering using batch corrected log2(n + 1)-transformed expression values shows that culture conditions affect the transcriptional profile of JX14P (Figure 5C). Samples distinctly cluster by their culture conditions, with clear differences in mRNA profiles of GBM cells grown in triculture vs. monoculture. Interestingly, JX14P grown in PSX and PDX appeared to have more similar transcriptional profiles with each other than when grown in triculture. A principal component analysis was performed to visualize the overall variance in the dataset and to assess the separation between culture conditions (Figure 5D). Triculture conditions separated from PDX and PSX culture conditions on PC1 with ~85% variation. These data suggest that the transcriptional profile variation in JX14P observed here is primarily driven by differences between the triculture condition and PDX/PSX culture conditions. There are no significant DEGs between monocultured JX14P cells in PDX media versus PSX media (Figure S3). On the other hand, several significant DEGs were identified in the PDX cells exposed to NHAs and macrophages compared to the monocultured PDX cells grown in either PDX media (Figure 5E) or PSX media (Figure 5F). Genes upregulated in the triculture model were largely related to stemness and hypoxia. Interestingly, several genes downregulated in the triculture model were related to interferon signaling.

## 3. Discussion

GBM exhibits both inter- and intra-tumoral heterogeneity. Numerous non-cancerous cell types exist within the TME and there is a dearth of preclinical in vitro models that accurately recapitulate cellular interactions of tumor cells with stromal and immune cells [23,24,26]. Most in vitro preclinical models fail to incorporate both stromal and immune cells in serum-free conditions. It is well established that the addition of serum to culture media causes BTICs to lose stem-like properties with reduced tumor-formation potential [24,27]. Additionally, glioma models grown in serum typically have increased chemo/radiation sensitivity. In this study, we identify a serum-free culture medium that supports the long-term growth of THP-1 monocytes and NHAs. When the described individual media are combined at a 1:1:1 ratio to generate PSX media, growth of all three cell types is supported in direct-contact triculture [23]. Importantly, all of this can be achieved while maintaining BTIC stem-like properties. We used multiple stemness surrogate assays, including ELDA, to determine the effects of different media on the stem cell population. In the GBM PDX lines tested, there was no reduction in stem-cell frequency for cells grown in PSX media relative to PDX media; however, there was a significant reduction in stem-cell frequency when cells were grown in serum-containing media relative to the other two media conditions.

We highlight the importance of serum-free media alternatives because the canonical astrocyte cell culture method has been relied upon with minor alterations since its establishment [28]. Standard macrophage and astrocyte protocols utilize FBS as an efficient growth factor source to promote cellular proliferation and survival. However, several studies have revealed that FBS contains significant levels of lipopolysaccharides and extracellular vesicles, both of which largely alter astrocytic biology and phenotypic outcome [14,15,29]. Aside from these additives, cattle sources of FBS differ greatly amongst batches due to various factors such as diet and herd diversity [14,29]. Further, many components in serum are unable to cross the BBB, and astrocytes typically remain quiescent in vivo unless in the presence of pathological insult (i.e., tumor cells) [7,14,15]. Tumor–cell interaction gives rise to TAAs that take on a reactive astrocyte phenotype, which is accompanied by increased levels of astrocyte differentiation markers such as GFAP [7,14,30]. For tumor–non-tumor cell studies, it is important to model this switch from quiescent to activated; however, this is difficult in the presence of FBS because it induces astrocytic differentiation without exposure to pathological mediators [14,15]. Comparable to other studies, we observed highest GFAP expression in FBS-exposed astrocytes [30]. Additionally, we observed FBS-exposed astrocytes lack the defined star shape characteristic to astrocytes seen in vivo [31,32]. In terms of functionality, astrocytes demonstrate excitability through intracellular Ca^2+^ changes rather than electrical excitability like neurons. The flux in Ca^2+^ levels is typically coupled with release of ATP or glutamate and increased calcium concentrations are also associated with reactive astrogliosis [14,33]. Therefore, we utilized this functionality aspect to compare basal intracellular calcium changes in astrocytes grown in SFA, PSX, and FBS. We predicted resting astrocytes would exhibit little change in calcium flux under basal conditions. SFA-grown NHAs had the lowest basal intracellular Ca^2+^ flux upon addition of sham stimuli (PBS), demonstrating the closest physiological relevance to a resting astrocyte. FBS conditions resulted in the highest basal intracellular Ca^2+^ flux, indicating FBS prematurely activates astrocytes without stimuli. Though PSX conditions induced a spike greater than SFA conditions at basal levels, we would expect that astrocytes may adopt a reactive state in the presence of GBM cells and macrophages, which is the primary purpose of PSX maintenance media. Upon addition of ATP, we see a significant increase in intracellular calcium flux amongst all groups, indicating that astrocytes in each media condition are responsive to the addition of ATP stimuli. However, it may be argued that FBS conditions induce artificial results that are exacerbated compared to how astrocytes might respond in vivo.

Current widely utilized macrophage polarization methods are suboptimal and do not accurately recapitulate the function or heterogeneity of TAMs. In vitro TAM models typically contain up to 10% FBS and are generated by polarizing resting macrophages using interleukins such as IL-4, IL-10, and IL-13 towards an M2-like phenotype. Unfortunately, this classic method of macrophage polarization is not representative of TAM function in vivo. While TAM function and phenotype are heterogeneous, THP-1 macrophage response to LPS and IFNγ stimulation is predictable. In this manuscript, we demonstrated that THP-1 monocytes respond to LPS and IFNγ stimulation by upregulating IL-1β on the transcriptional level and HLA-DR and CD80 on the cell surface. Future studies will characterize the phenotype of BTIC-polarized macrophages relative to classically polarized macrophages.

To assess if differential gene expression is observed in monocultured PDX cells versus PDX cells exposed to NHAs and macrophages, we utilized a custom 350-gene NanoString panel, as described previously [34]. The custom chip characterizes several canonical phenotypes in GBM, including but not limited to cell cycle, hypoxia, and GBM molecular subtypes (i.e., proneural vs. mesenchymal). No sigDEGs between monocultured JX14P cells in PDX media vs. PSX media were identified, further supporting our stemness-validation metrics. Contrastingly, several sigDEGs were identified in the PDX cells exposed to NHAs and macrophages compared to the monocultured PDX cells. Genes upregulated in the triculture model were largely related to stemness and hypoxia. Interestingly, several genes downregulated in the triculture model were related to interferon/STAT1 signaling. The current significant DEG analysis serves as a general overview of important gene signatures and opens new avenues to explore in future studies.

GBM is a highly heterogenous tumor and remains one of the most difficult to accurately model in vitro. Even when using PDX cells, monocultures do not represent the contributions of other cell types or the tumor microenvironment. TAMs and astrocytes secrete factors that promote tumoral growth, invasion, chemoradioresistance, and angiogenesis. TAAs secrete a variety of immunomodulatory factors such as transforming growth factor ß (TGFß), interleukin-6 (IL-6), interleukin-19 (IL-19), vascular endothelial growth factor (VEGF), insulin growth factor-1 (IGF-1), CCL2, and monocyte chemotactic protein-4 (MCP-4) [35,36]. Interestingly, we did find that PDX:SFA based co-culture of GBM PDX cells with NHA could enhance PDX proliferation capacity, which would support astrocyte-mediated growth signaling in the PDX (Figure S4). Collectively, these secreted molecules could likely promote tumor invasion, proliferation, and growth, and further drive the immunosuppressive phenotype of TAMs. Future studies will focus on investigating these processes in our triculture model.

Though we predict this model will greatly aid future GBM studies, there are several limitations that need to be acknowledged. Firstly, the use of immortalized astrocytes and monocytes over primary lines is inherently disadvantageous, but we are confident our protocol can be easily adapted. Additionally, the lack of other relevant cell types such as endothelial cells poses another obstacle as these cells are largely involved in the generation of blood vessels that supply tumors. To mitigate this, future studies will focus on adding additional cell types into the existing triculture model. In addition, one could imagine generating heterogenous tumor tricultures in which two (or more) different tumor lines (differentially labeled) are co-cultured with astrocytes and macrophages to even better replicate intra-tumoral heterogeneity. We also acknowledge that the model presented here is two-dimensional, which does not recapitulate the surrounding extracellular matrix that significantly contributes to communication dynamics. However, we are actively advancing our model to include a three-dimensional bioprinted matrix to better represent the tumor microenvironment. Most importantly, these drawbacks are temporary and are actively being improved as we continue to utilize fewer animal models while still efficiently representing disease states.

To model the intricate interactions between multiple cell types, certain measures must be taken to achieve these specific cellular states, on which we have presented our efforts herein. To the best of our knowledge, this is the first report of a serum-free triculture model system that supports the growth and function of these three human-derived cell types while maintaining GBM stem-like properties. Future studies will be required to further optimize the system by incorporating a 3D extracellular matrix and vascular components and profiling the phenotypes of GBM and the parenchymal cells to identify therapeutic vulnerabilities. The novel approach to in vitro GBM modeling presented herein enables the implementation of high-throughput screening in multi-cellular systems.

## 4. Materials and Methods

### 4.1. PDX and Stromal Cell Lines

JX14 parental (JX14P) was originally obtained from Mayo Clinic (Rochester, NY, USA) and maintained in the UAB Brain Tumor Model Core and was approved for use by a UAB IRB-approved protocol (IRB-300002910). JX14 (i.e., “GBM14”) was initially a recurrent, IDH-wildtype glioblastoma from a male patient. Information regarding mutational burden and copy number variants for JX14 is available on cBioPortal [37]. The matched acquired radiation-resistant PDX line (JX14P-RT) development has been previously described [38,39]. PDX authentication was confirmed using short tandem repeat (STR) analysis. THP-1 monocytes were purchased from ATCC (Manassas, VA, USA, Cat. #TIB-202) and NHA were purchased from Lonza (Basel, Switzerland, Cat. #CC-2565).

### 4.2. Media Formulations

The PDX Media (Table 1), SFA Media (Table 2), X-VIVO Media (Table 3), 10% FBS Media (Table 4), RPMI 1640 Media (Table 5), and PSX Maintenance Media (Table 6) formulations are provided.

### 4.3. Media Transition Instructions

The serum-containing to serum-free transition approaches for astrocytes (Table 7) and macrophages (Table 8) are indicated.

### 4.4. THP-1 Macrophage Polarization

THP-1 differentiation (Table 9) and polarization (Table 10) approaches are indicated.

### 4.5. Viability (Growth) Assay

A total of 1000 PDX cells were seeded in a Geltex-coated 96-well plate and allowed to grow for 1 week in their respective media for 7 days. Media was refreshed on day 4 with 1/3 of the total volume. Viability was assessed using CellTiter-Glo 2.0 (Promega, Madison, WI, USA, Cat: G9241) on day 7. In parallel, 100,000 PDX cells were seeded in Geltrex-coated T-25 flasks and grown for 1 week. All media groups were then plated into a Geltrex-coated 96-well plate in native PDX media and assessed for viability in the same manner on day 7. Statistical analysis was performed using a one-way ANOVA with multiple comparisons (*n* = 4 per group).

A total of 500 NHA cells were seeded in a Geltrex-coated 96-well plate and placed in their respective media for 14 days. Viability was assessed on day 7 using CellTiter-Glo 2.0. Every 4 days, 100% of the media volume was completely. Statistical analysis was performed using a one-way ANOVA with multiple comparisons (*n* = 6 per group).

THP-1 monocytes were grown for 1 week in RPMI 1640, X-VIVO 15, or PSX media. A total of 2500 cells from each condition were seeded in 100 µL/well in an uncoated 96-well plate. CellTiter-Glo assay was performed every 24 h using CellTiter-Glo 2.0, starting at t = 0. Luminescence was normalized to t = 0 for each condition. Statistical analysis was performed using a repeated-measures ANOVA with multiple comparisons (*n* = 8 per group).

### 4.6. Sphere Formation Assay

A total of 100,000 PDX cells were seeded into T-25 flasks and grown for 1 week. A total of 1000 single cells from each media group were then seeded into a 96-well plate in native PDX media and assessed for sphere-formation capability on day 7. Sphere formation was assessed and counted by imaging the plate on the Cytation5 Multimode Reader (4× Ph/0.1 NA, 4× BF/0.13 NA). Spheres were counted using automated Gen5 software (Agilent, Santa Clara, CA, USA) with manually preset circularity and size parameters of 40–150 µm. Statistical analysis was performed using a one-way ANOVA with multiple comparisons (*n* = 3 per group).

### 4.7. Western Blot

A total of 100,000 PDX cells were seeded into 6-well plates and grown in their respective media for 7 days. Cells were collected and lysed in mammalian protein extraction reagent (mPER) (ThermoFisher, Waltham, MA, USA, Cat. #78501), Halt Protease inhibitor (ThermoFisher, Waltham, MA, USA, Cat. #1861279), Phosphatase inhibitor (Sigma, St. Louis, MO, USA, Cat. #P5726-5ML), Halt Phosphatase inhibitor (ThermoFisher, Waltham, MA, USA, Cat. #1861277). Cells were spun at 14,000 RPM for 15 min at 4 °C and supernatants were collected. A Bicinchoninic acid (BCA) assay was performed to determine protein concentration. OLIG2 (Abcam, Waltham, MA, USA, ab109186), GFAP (ThermoFisher, Waltham, MA, USA, Cat #: MA5-12023), ALDHL1/L2 (Abcam, Waltham, MA, USA, ab177463), and β-actin (Santa Cruz, Dallas, TX, sc47778) were detected at a 1:1000 concentration, and an HRP-conjugated secondary antibody was incubated at a 1:5000 concentration (Jackson Labs, Bar Harbor, ME, USA, Code: 115-035-166). This experiment was performed in biological triplicate. Protein concentrations were normalized via densitometry to β-actin control and assessed statistically using a one-way ANOVA with multiple comparisons (*n* = 3 per group).

### 4.8. Extreme Limiting Dilution Assay (ELDA)

A total of 100,000 PDX cells were seeded into 6-well plates and grown in their respective media for 7 days. Cells were collected and dissociated into single cells with Accutase. To remove existing multicellular spheres, cells were passed through a 70 µm filter. Cells were seeded at the following densities per well: 1.25, 2.5, 5, 10, 20, and 40. Cells were grown for 14 days before fixation with 0.8% paraformaldehyde for 30 min at 4 °C. Cells were then stained with AF488-conjugated wheat germ agglutinin (2.5 µg/mL) for 24 h. Plates were then imaged on the Cytation5 Multimode Reader (Agilent, Santa Clara, CA, USA). AF488-positive spheres were counted using automated Gen5 software (Agilent, Santa Clara, CA, USA) with manually preset size parameters of >100 µm. ELDA analysis was performed using the software package developed by Hu et al. [40].

### 4.9. Immunofluorescence

NHAs were seeded onto Geltrex-coated coverslips at a concentration of 6 × 10^5^. Cells were fixed with a 1:1 ratio of 4% PFA and 0.05% glutaraldehyde for 15 min. Cells were quenched for 10 min with 100 mM NH4Cl and then permeabilized with 0.01% triton-X for 5 min. NHAs were probed with primary antibodies against GFAP (ThermoFisher, Waltham, MA, USA, Cat #: MA5-12023) at a concentration of 1:500. Cells were incubated with secondary antibodies conjugated to AF-488 (ThermoFisher, Waltham, MA, USA, Cat # A32731) or AF-647 (ThermoFisher, Waltham, MA, USA, Cat # A32733). Images were obtained on the Xcyto10 image cytometer (ChemoMetec, Allerod, Denmark) and were statistically quantified using XcytoView 1.1.11.0 software (ChemoMetec, Allerod, Denmark). Statistical analysis was performed using a one-way ANOVA (*n* = 3 per group).

THP-1 macrophages were seeded onto Geltrex-coated coverslips at a concentration of 1 × 10^5^. Cells were fixed with 4% PFA for 15 min. The cells were incubated with αCD80-BV711 and αHLA-DR-BV605 antibodies at a concentration of 1:1000. Afterwards, slides were washed 3× with PBS and incubated with DAPI and BlueMask at a concentration of 1:1000 for 30 m. Coverslips were then mounted onto XCyto10 two-sample slides. Images were obtained on an Xcyto10 Image Cytometer. Statistical analysis was performed using a one-way ANOVA (*n* = 4 per group).

PDX cells were seeded on a Geltrex-coated coverslip in a 24-well plate at a concentration of 100,000 cells per well. Cells were fixed with 4% PFA for 15 min. Cells were blocked with 2% BSA for 1 h and then stained with primary antibody at a concentration of 1:500 overnight at 4 °C. Cells were incubated with the secondary antibody at a concentration of 1:1000 for 1 h at RT and then incubated with DAPI and BlueMask at a concentration of 1:1000 for 30 m. Coverslips were then mounted onto Xcyto10 two-sample slides. Images were obtained using an Xcyto10 Image cytometer. Statistical analysis was performed using a one-way ANOVA (*n* = 4 per group).

### 4.10. NLS-mCherry-Expressing PDX Line Production

A total of 1,000,000 HEK293T cells were plated on a Geltrex-coated 10 cm^2^ dish. Cells were transfected with 2 µg of PsPAX2 (Addgene, MA, USA, Plasmid #12260), 4 µg of PMD2.G (Addgene, MA, USA, Plasmid #12259), and 6 µg of the NLS-mCherry vector (vectorbuilder: VB220505-1251vxc) for 24 h using the Lipofectamine 3000 reagent (Invitrogen, Waltham, MA, USA, Cat.#L3000015) in Opti-MEM (Invitrogen, Waltham, MA, USA, Cat.#11058). After 16 h, media was replaced with 4 mL of fresh PDX media. Media was collected and replaced every 24 h. The media was spun down at 2000 g for 10 m and the supernatant was collected and pooled. After 72 h of collection, the media was concentrated using Takara Biosciences Lenti-X Concentrator following the manufacturer’s protocol. The viral pellet was resuspended in 1 mL of PDX media. A total of 100 µL of virus was added to an Eppendorf tube containing 100,000 JX14 cells in a total volume of 500 µL with 8 µg/mL polybrene. A spinfection was performed by spinning the virus-cell mixture at room temperature for 45 min at 200 g. Afterwards, the supernatant was aspirated, and the cells were resuspended in 5 mL of fresh PDX media and plated in a Geltrex-coated T25 flask. The cells were expanded and subsequently flow-sorted for mCherry-positive cells.

The lentiviral vector used to overexpress NLS-mCherry in our study, pLV[Exp]-Puro-CMV + intron > NLS_mCherry, was constructed and packaged by VectorBuilder, USA. The vector ID is VB220505-1251vxc, which can be used to retrieve detailed information about the vector on vectorbuilder.com.

### 4.11. Calcium Assay

A total of 10,000 NHAs were plated on Geltrex in a black-walled 96-well plate. Cells were stained with Fluo-4 Direct™ Calcium Assay Kit (Invitrogen, Waltham, MA, USA Cat. #F10471) according to the protocol. Cells were imaged on a Cytation5 multimode imager. A baseline read was recorded for 25 s before PBS or 100 µM ATP dissolved in PBS was administered into one well. Images were taken every 5 s for 300 s. Relative fluorescence was obtained by normalizing each read to the averaged baseline. Statistical analysis was conducted using a two-way repeated-measures ANOVA and a multiple comparisons test.

### 4.12. Macrophage Digital PCR

A total of 500,000 THP-1 monocytes were plated and differentiated to macrophages in a 6-well plate. Afterwards, they were either maintained as resting or polarized towards an M1-like phenotype as previously described. RNA was extracted using a Qiagen, Venlo, The Netherlands, RNEasy mini kit (Qiagen, Venlo, The Netherlands, Cat# 74104). One milligram of RNA was reverse transcribed into cDNA using M-MLV Reverse Transcriptase (Promega, Madison, WI, USA, Cat# M1701) and then brought up to a total volume of 100 µL. IL-1ß (Hs01555410_m1) primers were conjugated to FAM dye and GAPDH (Hs00266705_g1) primers were conjugated to VIC dye. Digital PCR reactions consisted of 12 µL reaction mixture per well containing 3 µL of 4× Probe PCR master mix (Qiagen, Venlo, The Netherlands, Cat# 250102), 1.2 µL of 10× target primer conjugated to FAM, 1.2 µL of 10× GAPDH primer conjugated to VIC, 4 µL of 1:80 diluted cDNA, and 2.6 µL of PCR-grade water. Assembled reactions were transferred to QIAcuity 8.5 k 96-well nanoplates for partitioning using the standard priming protocol. dPCR was conducted in 40 cycles of 95 °C denaturation for 15 s and 60 °C annealing and elongation for 30 s. Partitions were imaged with 500 ms exposure time and with gain set to 6 for both target channels. Raw counts were normalized to GAPDH. Statistical analysis was performed using a two-way ANOVA (*n* = 4 per group).

### 4.13. Direct Triculture Experiments

A total of 25,000 THP-1 monocytes were plated into a 24-well plate with Geltrex-coated coverslips, differentiated into macrophages, and polarized towards a TAM-like state. Afterwards, 25,000 NHAs and 50,000 NLS-mCherry JX14 were added to the well and allowed to grow for an additional 72 h. Afterwards, cells were fixed and stained as previously described. Statistical analysis was performed using a one-way ANOVA (*n* = 4 per group).

### 4.14. CFSE-Retention Assay

NLS-mCherry cells were labeled with 5 µM of Carboxyfluorescein succinimidyl ester (CFSE) (Thermofisher, Waltham, MA, USA, Cat. #C34554). Afterwards 500 cells were plated in each well of a 96-well plate and grown in their respective media for 1 week. Images were obtained using a Cytation5 cell imaging multimode reader at 4× magnification. The % of CFSE-positive cells was calculated as a ratio of the total number of mCherry-positive cells. Statistical analysis was performed using a one-way ANOVA (*n* = 6–10 per group).

### 4.15. NanoString nCounter^®^ Analysis

A total of 500,000 PDX cells were seeded into 6-well plates either in monoculture or indirect triculture and grown in their respective media for 72 h. For triculture experiments, astrocytes and macrophages were grown in the upper well of a transwell insert with 3.0 µm pores (Corning, New York, NY, USA, Cat. #353091). RNA isolation was performed using 0.5 mL TRIzol Reagent (Invitrogen, Waltham, MA, USA, Cat. #15596018). nCounter data was exported to RStudio (v 4.3.2) where it was normalized and filtered as previously described [34].

The LIMMA package (v3.58.1) in R (v 4.3.2) was used to identify differentially expressed (DE) genes in the JX14 cells across various culture conditions and passage groups with passage number as a covariate. A design matrix was constructed using the model.matrix function, followed by fitting a linear model with lmFit(). Pairwise contrasts of interest were defined with makeContrasts() and applied to the model using constrasts.fit() and eBayes() to moderate variance. Differentially expressed genes (DEGs) for all contrasts were extracted using topTable(), with *p*-values adjusted by FDR method (q).

Differentially expressed genes, q < 0.05 and |Log2 Fold Change| >1, are displayed on volcano plots using the EnhancedVolcano package (v1.20.0). Heatmaps were generated using pHeatmap (1.0.12) on log2(n + 1)-transformed counts. PCA plots were generated with PCAtools v(2.15.0) on all genes captured by the NanoString assay.

### 4.16. Xcyto10 Quantitative Image Cytometer

Samples were imaged on a Xcyto10 fluorescence microscope (ChemoMetec, Allerod, Denmark) equipped with 4 high-power LEDs (405, 488, 535, and 635 nm) for fluorescence and 1 UV LED (365 nm) for transmitted light imaging, as well as 36 fluorescent channels employing 9 emission filters using ChemoMetec (Allerod, Denmark) 4×/0.2 NA and 20×/0.5 NA objective air lens. Samples were acquired with a CCD 2.8 MP camera (1920 × 1440 pixels), pixel size: 4.54 μm. Statistical analysis was performed using a one-way ANOVA (*n* = 4 per group).

### 4.17. Cytation5 Cell Imaging Multimode Reader

Samples were imaged on an Agilent Cytation5 fluorescence widefield microscope equipped with a xenon flash lamp (Agilent, Santa Clara, CA, USA), using an Olympus 4×/0.13 NA air lens, 4× Phase/0.1 NA air lens, 20×/20× Phase 0.45 NA air lens. EGFP was detected using a 469/35 nm EX (Agilent, Santa Clara, CA, USA), 525/39 nm EM (Agilent), and 497 nm dichroic mirror (Agilent, Santa Clara, CA, USA). Texas Red was detected using a 586/15 nm EX (Agilent, Santa Clara, CA, USA), 647/57 nm EM (Agilent, Santa Clara, CA, USA), and 605 nm dichroic mirror. The samples were acquired with a Sony IMX 264 CMOS WFOV camera (1992 × 1992 pixels), pixel size: 3.45 μm. Statistical analysis was performed using a one-way ANOVA (*n* = 4 per group).

### 4.18. Statistical Analysis

The specific methods of corresponding items are described above. Statistical analysis was conducted using GraphPad Prism 10.4.1 unless otherwise stated. Statistical analysis was performed using one-way ANOVA followed by post hoc Tukey’s multiple comparisons test, unless otherwise stated. All experiments were performed in at least three technical replicates and repeated independently at least two times. The number of observations per individual experiment is presented in the respective figure legend description. These data are presented as mean ± SD. * *p* < 0.05, ** *p* < 0.01, *** *p* < 0.001, **** *p* < 0.0001.

## 5. Conclusions

Despite decades of research, patient survival in GBM remains dismal. The drug development process has been hindered due to the lack of high-fidelity preclinical models of GBM. Current in vitro models can be improved to better represent the cellular heterogeneity of the tumor microenvironment. Cells such as astrocytes and macrophages are known to substantially promote tumor progression but are typically excluded from in vitro models. To advance existing preclinical in vitro models, the complex interactions between major cell types comprising the GBM tumor microenvironment must be replicated. In this study, we highlight a novel method by which we can culture NHAs, macrophages, and GBM PDX cells in a serum-free environment that preserves function of all cell types and maintains GBM stem-like properties. Future studies will utilize this model to identify targetable pathways that can lead to the development of therapeutics that may one day enter the clinic and improve patient outcomes in GBM.

## Figures and Tables

**Table 1:** Composition of PDX Media.

Patient-Derived Xenograft (PDX) Media	Per 500 mL	Product Information
DMEM-F12 50/50	500 mL	Corning, New York, NY, USA, Cat. #10-090-CV
B27 Supplement (50×)	10 mL	Gibco, Waltham, MA, USA, Cat. #17504044
Penicillin-streptomycin (100×)	5 mL	Corning, New York, NY, USA, Cat. #30-001-CI
Sodium Pyruvate (1 mM)	5 mL	Corning, New York, NY, USA, Cat. #5000CI
Human EGF	10 µg	Gibco, Waltham, MA, USA, Cat. #PHG0311
Human FGF	10 µg	Gibco, Waltham, MA, USA, Cat. #PHG0261

**Table 2:** Composition of SFA Media.

Serum-Free Astrocyte (SFA) Media	Per 1 L	Product Information
DMEM-F12 50/50	500 mL	Corning, New York, NY, USA, Cat. #10-090-CV
Neurobasal	500 mL	Gibco, Waltham, MA, USA, Cat. #12349015
Penicillin-streptomycin (100×)	10 mL	Corning, New York, NY, USA, Cat. #30-001-CI
Sodium Pyruvate (1 mM)	4.16 mL	Corning, New York, NY, USA, Cat. #5000CI
Bovine Serum Albumin	100 mg	Sigma, St. Louis, MO, USA, Cat. # A9418-5G
N2 Supplement (100×)	10 mL	Gibco, Waltham, MA, USA, Cat. #17502048
N-Acetyl Cysteine	2.5 mg	ThermoFisher, Waltham, MA, USA, Cat. #160280250
Human EGF	20 µg	Gibco, Waltham, MA, USA, Cat. #PHG0311
Human FGF	20 µg	Gibco, Waltham, MA, USA, Cat. #PHG0261

**Table 3:** Composition of X-VIVO Media.

TheraPEAK^TM^ X-Vivo^TM^ Media	Per 1 L	Product Information
TheraPEAK™ X-VIVO™-15 Serum-free Hematopoietic Cell Medium	1 L	Lonza, Basel, Switzerland, Cat.#BEBP02-055Q
Penicillin-streptomycin (100×)	10 mL	Corning, New York, NY, USA, Cat.#30-001-CI
Sodium Pyruvate (1 mM)	10 mL	Corning, New York, NY, USA, Cat.#5000CI
Glutamax (100×)	10 mL	Gibco, Waltham, MA, USA, Cat.#35050-061
TheraPEAK™ X-VIVO™-15 Serum-free Hematopoietic Cell Medium	1 L	Lonza, Basel, Switzerland, Cat.#BEBP02-055Q

**Table 4:** Composition of 10% FBS Media.

10% FBS Media	Per 500 mL	Product Information
DMEM-F12 50/50	500 mL	Corning, New York, NY, USA, Cat. #10-090-CV
Fetal Bovine Serum	50 mL	Sigma, St. Louis, MO, USA, Cat.#F0926
Penicillin-streptomycin (100×)	5 mL	Corning, New York, NY, USA, Cat.#30-001-CI

**Table 5:** Composition of RPMI 1640 Media.

RPMI	Per 500 mL	Product Information
RPMI 1640 Medium with ATCC modification	500 mL	Gibco, Waltham, MA, USA, Cat# A1049101
Fetal Bovine Serum	50 mL	Sigma, St. Louis, MO, USA, Cat.#F0926
Penicillin-streptomycin	5 mL	Corning, New York, NY, USA, Cat.#30-001-CI

**Table 6:** Composition of Maintenance Media.

PSX Maintenance Media 1:1:1 *v*/*v*
PDX media
SFA media
TheraPEAK^TM^ X-VIVO^TM^ 15 media

**Table 7:** SFA Media Transition Schedule.

Serum-Free Astroctye (SFA) Media	Week	Notes
Coat vessel with Geltrex and seed 70% confluency. Change media ratio: 90% FBS native + 10% SFA.	1	Geltrex (Gibco, Waltham, MA, USA, Cat. #A15696-01) should be diluted to 0.12–0.18 mg/mL.
Change media ratio: 70% FBS native + 30% SFA.	2	Keep astrocytes on Geltrex during the transition stage.
Change media ratio: 50% FBS native + 50% SFA.	3	Change 70% of media every 3 days, adjust according to cell growth.
Change media ratio: 30% FBS native + 70% SFA.	4	Transition process can be expedited if cells are responding well.
Change media ratio: 10% FBS native + 90% SFA.	5	
Transition to 100% SFA media	6	

**Table 8:** X-VIVO Media Transition Schedule.

TheraPEAK^TM^ X-VIVO^TM^ 15 Media	Week	Notes
Plate cells at a concentration of 400k cells/mL. Change media ratio: 50% FBS native + 50% X-VIVO	1	Media changes every 2–3 days.
Change media ratio: 25% FBS native + 75% X-VIVO	2	Media changes every 2–3 days.
Change media ratio: 10% FBS native + 90% X-VIVO	3	Media changes every 2–3 days.
Change media ratio: 5% FBS native + 95% X-VIVO	4	Media changes every 2–3 days.
Change media to 100% X-VIVO	5	Media changes every 2–3 days.

**Table 9:** THP-1 Differentiation Protocol.

THP-1 Monocyte Differentiation to Macrophages	Day	Notes
Incubate THP-1 monocytes with 20 nM 12-O-Tetradecanoylphorbol-13-acetate (PMA) for 24 h.	1	Monocytes will become adherent and increase in size
Replace PMA-containing media with fresh media	2	THP-1 macrophages are more M1-like post differentiation, this rest period allows them to revert to a resting state.

**Table 10:** THP-1 Macrophage Polarization Protocol.

THP-1 Macrophage Polarization	Day	Notes
Incubate THP-1 macrophages with 20 ng/mL IFN-γ and 1 µg/mL lipopolysaccharide (LPS) for 24 h to achieve a pro-inflammatory (M1-like) state	3	IFN-γ (Gibco, Waltham, MA, USA, Cat. #300-02-500UG) LPS (ThermoFisher, Waltham, MA, USA, Cat. #00-4976-03)

## Data Availability

Array data has been deposited to EMBL-EBI ArrayExpress Archive (Accession # E-MTAB-14811). Code is available upon request.

## Supplementary Materials

The following supporting information can be downloaded at https://www.mdpi.com/article/10.3390/ijms26199335/s1.

## Abbreviations

The following abbreviations are used in this manuscript:

BBB	Blood–brain barrier
BTIC	Brain tumor-initiating cells
CFSE	Carboxyfluorescein succinimidyl ester
ELDA	Extreme limiting dilution assay
FBS	Fetal bovine serum
GBM	Glioblastoma
NHA	Normal human astrocytes
NPC	Neural progenitor cells
PDX	Patient-derived xenografts
PSX	PDX-SFA-X-VIVO media
SFA	Serum-free astrocyte
TAA	Tumor-associated astrocytes
TAM	Tumor-associated macrophages
TME	Tumor microenvironment
