# Supplementary material for: A Novel Serum-Free Triculture Model of Glioblastoma, Astrocytes, and Macrophages
Source: Int J Mol Sci. 2025 Sep 24;26(19):9335. doi: 10.3390/ijms26199335 (PMC12524639; doi:10.3390/ijms26199335)
Supplement: Supplementary file 1 [file ijms-26-09335-s001.zip › ijms-3843594-supplementary.pdf]

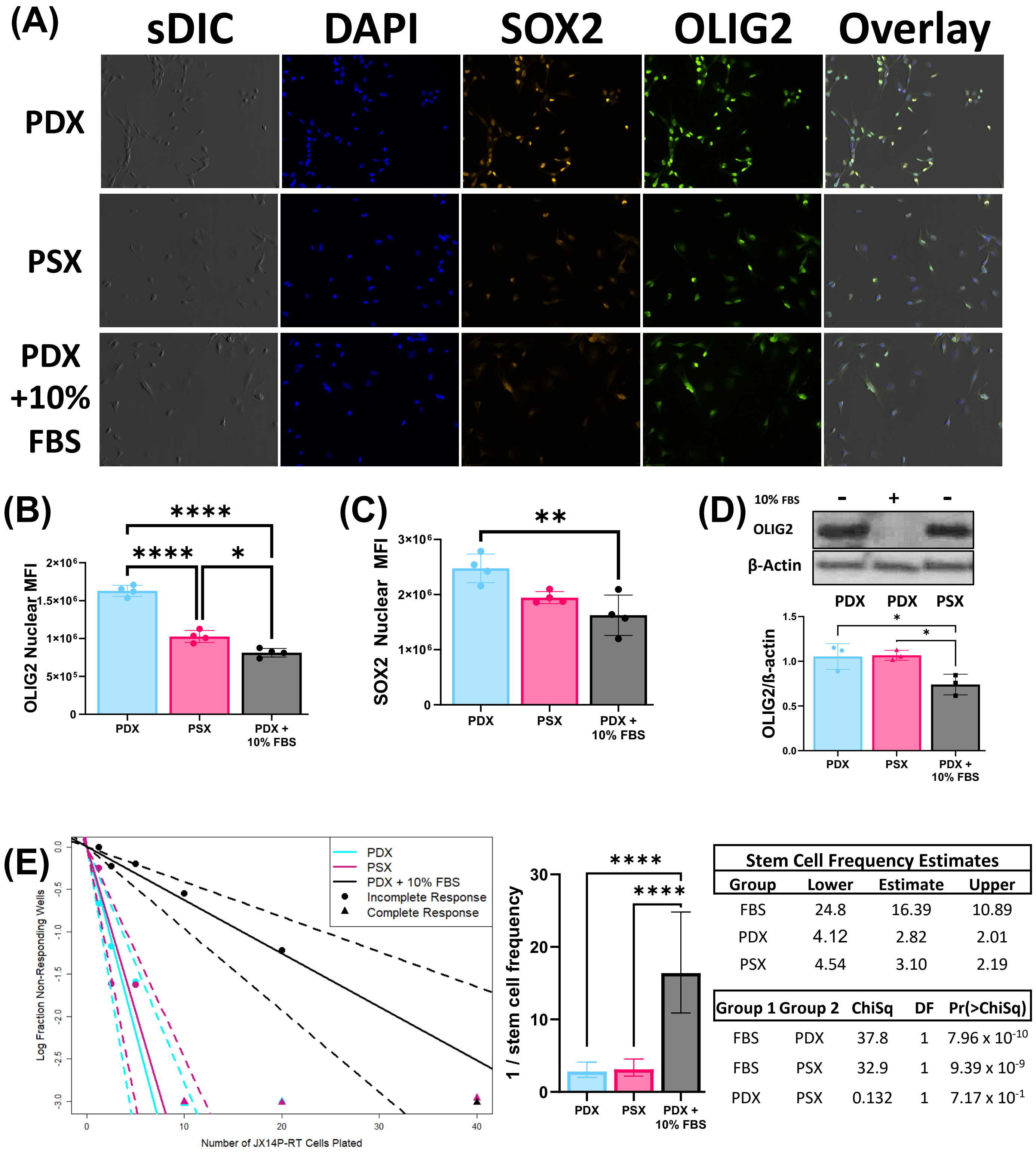

Stem Cell Frequency Estimates			
Group	Lower	Estimate	Upper
FBS	24.8	16.39	10.89
PDX	4.12	2.82	2.01
PSX	4.54	3.10	2.19

Group 1	Group 2	ChiSq	DF	Pr(>ChiSq)
FBS	PDX	37.8	1	7.96×10^{-10}
FBS	PSX	32.9	1	9.39×10^{-9}
PDX	PSX	0.132	1	7.17×10^{-1}

Supplementary Figure 1: PSX media maintains the stemness properties of JX14P-RT. (A) Representative images of JX14P-RT probed with stemness markers, SOX2 and OLIG2. **(B)** Quantification of OLIG2 nuclear MFI in respective medias. **(C)** Quantification of SOX2 nuclear MFI in respective medias. **(D)** Representative western blot of OLIG2 protein concentrations with quantification in JX14P-RT after growth in respective medias for 7 days. **(E)** Extreme limiting dilution assay (ELDA) performed on JX14 when grown in respective media. * $p < 0.05$, ** $p < 0.01$, **** $p < 0.0001$

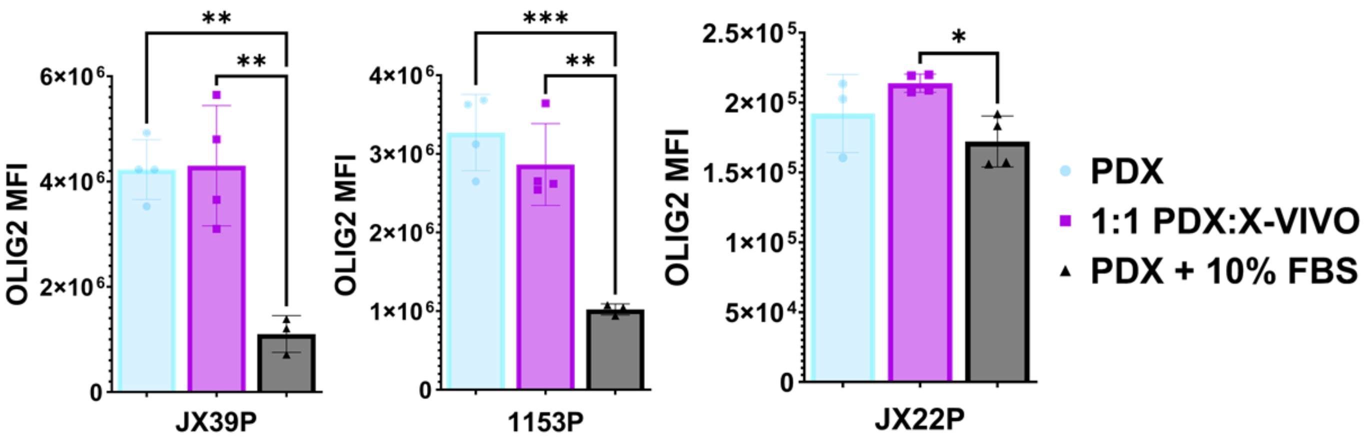

Supplementary Figure 2: Serum-free co-culture media maintains stemness marker expression in GBM PDX lines. 5×10^4 JX39P, 1153P, and JX22P were grown in either PDX, a 1:1 mixture of PDX and X-VIVO, or PDX media + 10% FBS for 72 hours on geltrex coated coverslips in a 24 well plate. Afterwards, they were fixed and stained for OLIG2 as described in the Materials and Methods. Images were obtained using an XCyto10 cytometer. One-way ANOVA with Tukey's post-hoc correction was performed for multiple comparisons testing (n=3-4). Quantification of OLIG2 MFI in the nucleus of GBM cell lines JX39P, 1153P, and JX22P is shown for PDX, 1:1 PDX:X-VIVO, and PDX supplemented with 10% FBS. For all three cell lines, there was no significant difference in OLIG2 expression for cells grown in PDX media and 1:1 mixture of PDX:X-VIVO. For JX39P and 1153, cells grown in PDX and 1:1 PDX:X-VIVO had significantly elevated levels of OLIG2 relative to those grown in media supplemented with FBS. JX22 showed a moderate increase in OLIG2 expression for cells grown in a 1:1 mixture of PDX:X-VIVO relative to FBS; however, there was no difference between cells grown in PDX and FBS media. Notably, JX22 had the lowest baseline expression of OLIG2. * $p < 0.05$, ** $p < 0.01$, *** $p < 0.001$

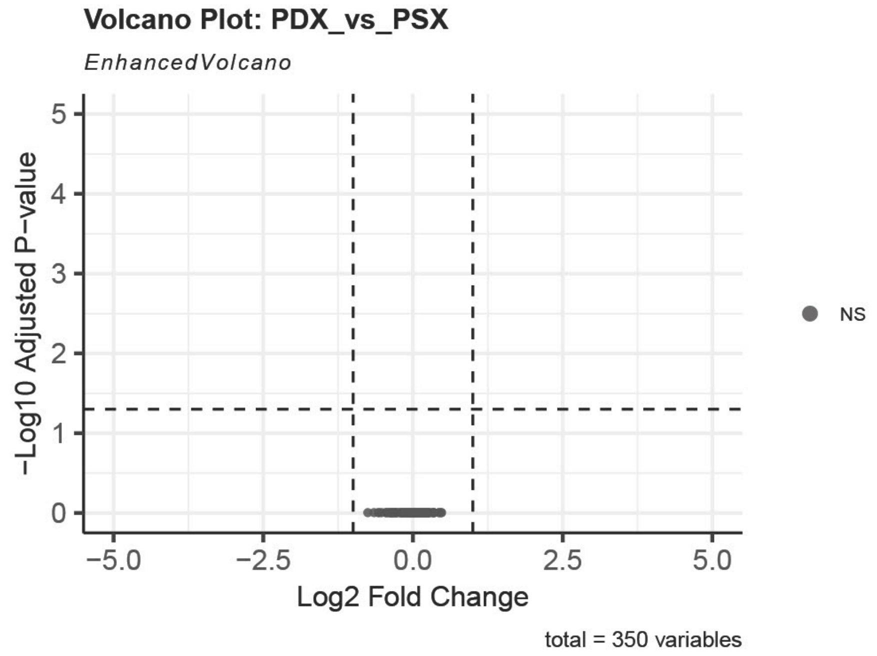

Supplementary Figure 3: DE Analysis of monocultured JX14 grown in PDX vs PDX. Differential Expression analysis showing no differential expression of genes in PSX monoculture relative to PDX monoculture with LFC cutoff of 1 and adjusted *p*-value cutoff of 0.05.

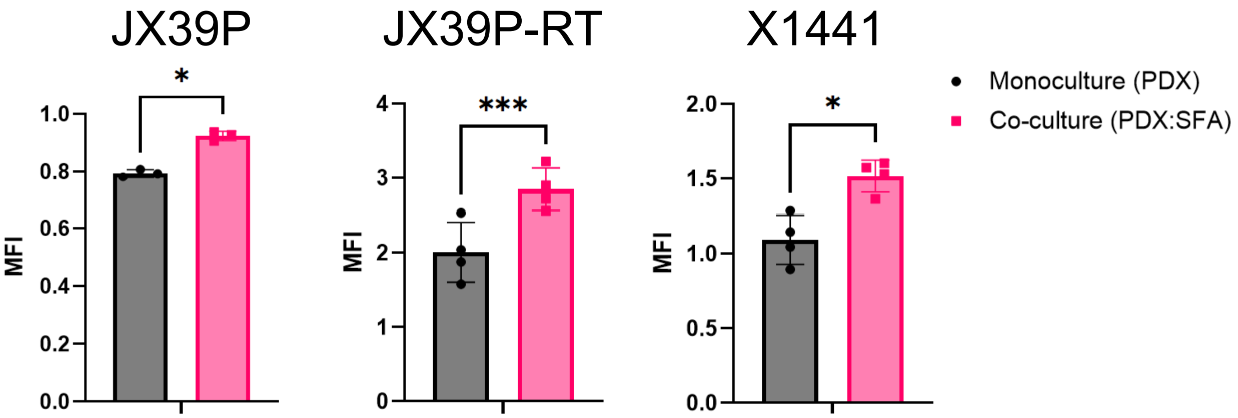

Supplementary Figure 4: Serum-free media co-culture of GBM PDX cells and NHA increases GBM PDX cell viability. mCherry-expressing JX39P, JX39P-RT, and X1441 cells were cultured in serum free PDX media as a monoculture or as a co-culture with NHA's using a 1:1 PDX:SFA mixture grown in a monolayer using geltrex coated plates. For each tumor line, 2500 PDX cells were plated with 2500 NHAs and after five days, the mean fluorescent intensity (MFI) for mCherry was measured using Cytation5 and plotted as Mean ± SD. Student's t test shown with * $p < 0.05$ and *** $p < 0.001$.
